# T cell-derived exosomes in tumor immune modulation and immunotherapy

**DOI:** 10.3389/fimmu.2023.1130033

**Published:** 2023-04-20

**Authors:** Qiujun Zhou, Shenyu Wei, Hui Wang, Yuanyuan Li, Shasha Fan, Yi Cao, Chenglei Wang

**Affiliations:** ^1^ Department of First Clinical Medical College, Zhejiang Chinese Medical University, Hangzhou, China; ^2^ Department of Hepato-Pancreato-Biliary Surgery, The Second Affiliated Hospital, Zhejiang University School of Medicine, Hangzhou, Zhejiang, China; ^3^ The First Affiliated Hospital of Zhejiang Chinese Medical University (Zhejiang Provincial Hospital of Chinese Medicine), Hangzhou, Zhejiang, China; ^4^ Center for Plastic & Reconstructive Surgery, Department of Dermatology, Zhejiang Provincial People’s Hospital (Affiliated People’s Hospital, Hangzhou Medical College), Hangzhou, Zhejiang, China

**Keywords:** T cell, exosome, tumor, cancer, immune modulation, immunotherapy

## Abstract

Exosomes are nanoscale vesicles secreted by most cells and have a phospholipid bilayer structure. Exosomes contain DNA, small RNA, proteins, and other substances that can carry proteins and nucleic acids and participate in communication between cells. T cells are an indispensable part of adaptive immunity, and the functions of T cell-derived exosomes have been widely studied. In the more than three decades since the discovery of exosomes, several studies have revealed that T cell-derived exosomes play a novel role in cell-to-cell signaling, especially in the tumor immune response. In this review, we discuss the function of exosomes derived from different T cell subsets, explore applications in tumor immunotherapy, and consider the associated challenges.

## 1 Introduction

Exosomes are nanoscale vesicles (30–160 nm) secreted by most cells and have a phospholipid bilayer structure (1). Exosomes contain DNA, small RNA, proteins, and other substances that can carry proteins and nucleic acids and participate in communication between cells (2). Previous studies have suggested that exosomes function as cellular garbage bags, eliminating redundant and non-functional cellular components (3). Recent studies have shown that exosomes are intercellular junctions that transport proteins, lipids, and nucleic acids to target cells, play a role in various biological processes (such as angiogenesis, antigen presentation, apoptosis, and inflammation), and can be used as diagnostic and therapeutic tools for diseases (4). It can also participate in various pathophysiological processes such as tissue repair, immune response, inflammation, and tumor growth and metastasis (5, 6).

T-lymphocytes are derived from pluripotent stem cells in the bone marrow (7). During the embryonic and primary stages of human life, pluripotent stem cells or proT cells in the bone marrow migrate to the thymus and mature into immunoactive T cells under the induction of thymus hormones (8). Intercellular communication is an essential hallmark of multicellular organisms and can be mediated through direct cell-cell contact or the transfer of secreted molecules (9). Increasing studies have shown that immune cells participate in cellular communication by secreting exosomes (10, 11). Among the immune cell-derived exosomes, T cell-derived exosomes have recently been reported to be involved in antitumor effects in cancer immunotherapy by mimicking the role of parental cells (12–15). The upregulation and downregulation of exosome production by T cells is a new method for regulating the immune response to tumors (16). Therefore, fully exploiting the characteristics of T cell-derived exosomes can effectively treat tumors. In this review, we summarize the pathogenesis and secretion of exosomes and describe the role of T cell-derived exosomes in tumor immune regulation and the application of T cell-derived exosomes in tumor immunotherapy to provide new ideas for the future treatment of cancers.

## 2 Biogenesis and secretion of exosomes

**Figure 1:**
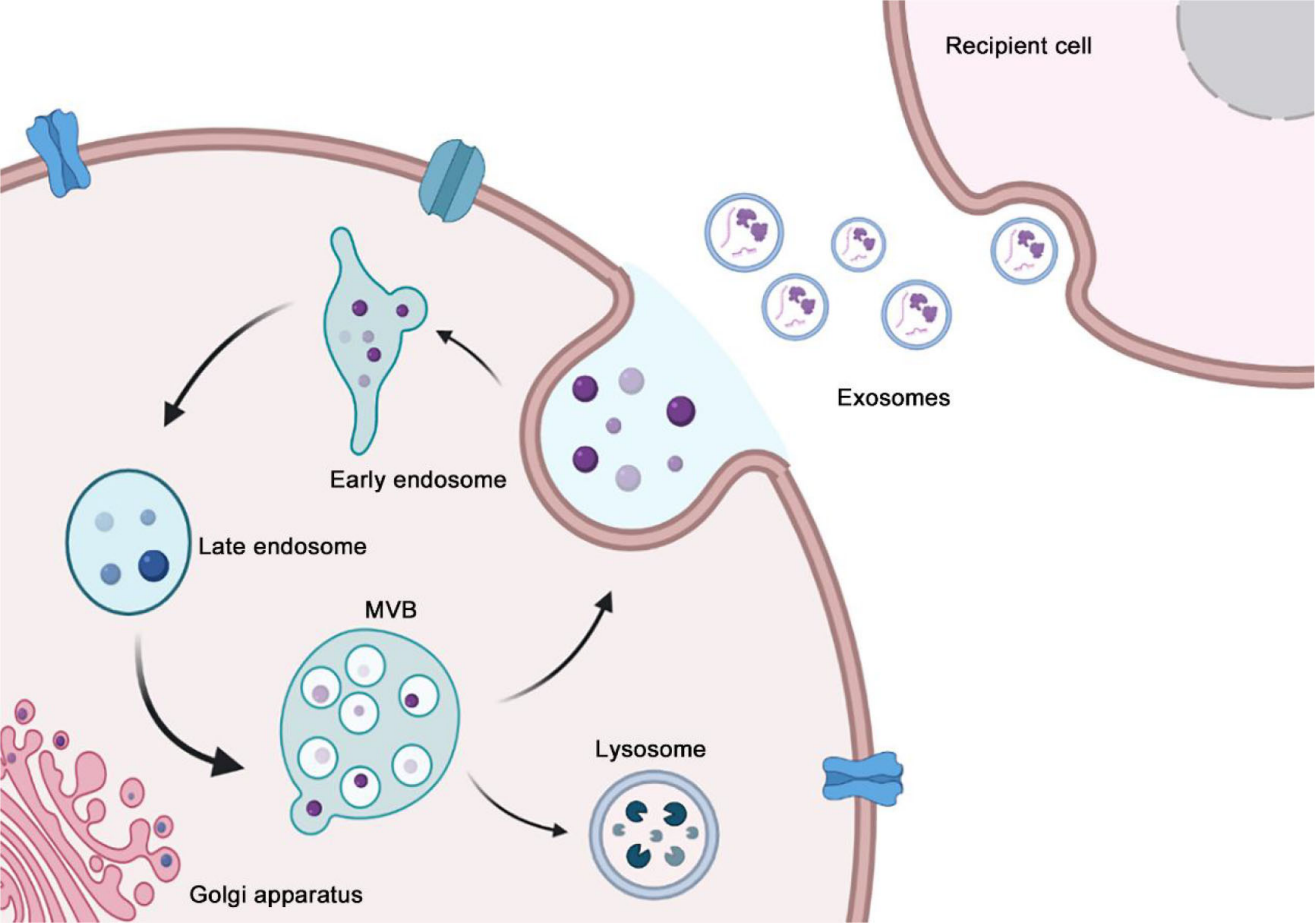
The process of exosome biogenesis and secretion. The biogenesis of exosome begins at early endosome formation through endocytosis at the plasma membrane, and then the invagination of the plasma membrane of LSEs forms ILVs that are ultimately secreted as exosomes. In the end, MVBs fuse with the plasma membrane to release exosomes. Ectosome originates from the outward budding and fission of the plasma membrane, subsequently, the nascent ectosomes are released into the extracellular space.

Exosomes are intraluminal vesicles (ILVs) formed by inward budding of the endosomal membrane during maturation of multivesicular bodies (MVBs). Subsequently, MVBs fuse with the plasma membrane to release the contained ILVs as exosomes or fuse with lysosomes or autophagosomes for degradation (17) (**Figure 1**). Various sorting mechanisms are involved in different steps of exosome formation (18). First, the limited membrane regions of MVBs are generally referred to as the dispersed microdomains. The formation of the cluster microdomain and the external mechanical action promote membrane budding, followed by the division of the plasma membrane into the extracellular medium or the limiting membrane of MVBs into the MVB lumen. Currently, the mechanism of exosome formation is well understood, and the subunits involved in the endosomal sorting complex required for transport (ESCRT) play an important role (19, 20). When ILVs enter the lumen of MVBs, the involvement of ESCRT-III is required to varying degrees; however, the processes of inclusion aggregation and membrane budding are not entirely dependent on ESCRT (21, 22). ESCRT-independent pathways have also been identified as alternative mechanisms and may coexist with ESCRT-dependent machinery in the formation of MVBs and sorting of internalized cargo (23, 24). Exosome production is complex and often depends on the host and the type of parent cell as well as other stimuli received by the cell. These inclusions participate in the germination, fission, and release of exosomes through progressive aggregation (25). In addition, the properties and content of exosome inclusions are specific and are often influenced by the physiological or pathological state of the maternal cell, stimuli that regulate their production and release, and molecular mechanisms that facilitate their production (2).

## 3 T cell-derived exosomes in tumor immune modulation

Similar to other cells, T cells produce exosomes that reflect their characteristics, such as directly killing target cells, assisting or inhibiting B cells to produce antibodies, responding to specific antigens and mitogens, and producing cytokines, thereby creating an optimal microenvironment for immune cell function in paracrine and autocrine forms (26). T cell-derived exosomes can activate other immune cells, suppress immune responses, and participate in the licensing of antigen-presenting cells (APCs) (26). In a recent study, researchers attached interleukin (IL)-2 to the transmembrane domain of platelet-derived growth factor receptor *via* a flexible linker and then incorporated the gene into lentiviruses for Jurkat T cell infection. The infected Jurkat T cells then secreted IL-2-exosomes, which showed significant changes in the expression of miR-181a-3p and miR-223-3p in IL-2-exosomes relative to untreated exosomes. miRNAs increase the activity of CD8^+^ T cells and decrease the expression of programmed death ligand 1 (PD-L1) in melanoma, resulting in increased sensitivity to CD8^+^ T cell-mediated cytotoxicity (27). T cells can regulate the release of distinct exosome subpopulations depending on their activation status (28). In the following sections, we discuss the role of different T cell subsets in tumor immunomodulation (**Table 1**).

**Table 1:** Role of T cell-derived exosomes in immune modulation.

The origin of exosomes	Mechanism of action	Content	Reference
CD8^+^ T cells	Attenuating PD-L1-induced suppression of tumor-specific cytotoxic T cell activity	PD-1	(32)
CD8^+^ T cells	Associated with the expression of immune checkpoint receptors on the surface of CD8^+^ T cells	uPAR	(36)
CD8^+^ T cells	Inhibited antitumor effect by decreasing the MHC-I in DCs and CD8^+^ T cell activity	LFA-1	(38)
CD8^+^ T cells	Limiting estrogen-driven development of UCEC *via* regulation of the miR-765/PLP2 axis	miR-765	(39)
CD8^+^ T cells	Mediating depletion of mesenchymal tumor stromal cells	–	(14)
Exhausted CD8^+^ T cells	Impairing the anticancer function of normal CD8^+^ T cells	lncRNAs	(40)
CD8^+^ T cells	Activating ERK and NF-κB pathways to induce melanoma metastasis	FasL	(41)
CD4^+^ T cells	Inducing CD8^+^ T cell-mediated antitumor responses	miR-25-3p, miR-155-5p, miR-215-5p, and miR-375	(43)
CD4^+^ T cells	Inhibiting CD8^+^ cytotoxic T lymphocyte responses and antitumor immunity in melanoma	LFA-1	(44)
CD4^+^ T cells	Involving in the regulation of humoral immunity	CD40L	(45)
Tregs	As a potential non-invasive tumor and immune cell biomarkers in HNSCC	–	(54)
Tregs	Resulting the production of a tolerogenic phenotype in DCs	miR-150-5p and miR-142-3p	(55)
CD8^+^ CD25^+^ Tregs	Inhibiting DC-induced cytotoxic T lymphocyte responses and antitumor immunity	–	(56)
Tregs	Promoting the expression of M2 macrophage markers	–	(57)
Tregs	Inhibiting the proliferation of CD4^+^ T cells	miR-146a-5p	(63)

### 3.1 CD8^+^ T cell-derived exosomes

CD8^+^ T cells are cytotoxic T lymphocytes (CTLs), a subset of white blood cells that secrete various cytokines to specifically kill target cells. It can remove virus-infected cells, tumor cells, and other antigenic substances and is an important defense line of antiviral and antitumor immunity (29). An increasing number of studies have revealed that CD8^+^ T cell-derived exosomes mediate information exchange between immune cells and tumor cells, thereby regulating tumor development. CD8^+^ CTLs fully activated by tumor antigens enhance the activation of low-affinity CD8^+^ T cells by secreting exosomes, thus participating in the tumor killing process (30, 31). For instance, Qiu et al. (32). found that programmed cell death 1 (PD-1), which is widely expressed in tumor-infiltrating lymphocytes of triple-negative breast cancer (TNBC) and is significantly associated with poor prognosis of TNBC (33, 34), can be secreted by activated T cells on the surface of exosomes, interacting remotely with PD-L1 on the cell surface or exosomes, and restoring tumor surveillance by attenuating PD-L1-induced suppression of tumor-specific cytotoxic T cell activity. In another clinical study, considering the effect of urokinase-type plasminogen activator (uPAR) signaling on tumors (35), Porcelli et al. collected blood samples from 71 patients with metastatic melanoma treated with immune checkpoint inhibitors (including responders and non-responders) and analyzed CD8^+^ T cell-derived uPAR^+^ exosome levels. The results of this study indicated that patients with immune checkpoint inhibitor-resistant melanoma had low levels of CD8^+^ T cell-derived uPAR^+^ exosomes in their blood (36). These findings suggest that CD8^+^ T cell-derived uPAR^+^ exosomes are associated with the expression of immune checkpoint receptors on the surface of CD8^+^ T cells, which is a direction for future research. The above studies provide a potential therapeutic strategy for modifying the exosome surface with membrane-bound inhibitory immune checkpoint receptors to attenuate the suppressive tumor immune microenvironment. Interestingly, CD8^+^ T cell-derived exosomes can also be endocytosed by APCs, cells in the body that can ingest, process, and transfer antigen information to induce the immune response of T and B cells (37), *via* pMHC-I/TCR interactions, and inhibit antigen-specific dendritic cell (DC)-mediated indirect CD8^+^ CTL responses (38). Specifically, exosomes derived from activated CD8^+^ T cells inhibited antitumor effects by decreasing MHC-I in DCs and CD8^+^ T cell activity in melanoma models (38). In addition to participating in the regulation of tumor growth by mediating information exchange between immune cells, CD8^+^ T cell-derived exosomes directly inhibit tumor progression. For example, Zhou et al. found that CD45RO^-^CD8^+^ T cell-derived exosomes released more miR-765 than CD45RO^+^CD8^+^ T cells. These exosomes miR-765 derived from CD45RO^-^CD8^+^ T cells limit estrogen-driven development of uterine corpus endometrial cancer (UCEC) *via* regulation of the miR-765/proteolipid protein 2 (PLP2) axis (39). Additionally, CD8^+^ T cells can inhibit tumor progression by exosome-mediated depletion of mesenchymal tumor stromal cells, in addition to their conventional direct cytotoxicity against tumor cells (14). The above studies support the idea that CD8^+^ T cell-derived exosomes are involved in the inhibition of tumor progression. However, CD8^+^ T cell-derived exosomes play a double-edged sword in tumorigenesis and development.

Wang et al. found that exosomes derived from exhausted CD8^+^ T cells can be taken up by normal CD8^+^ T cells and impair their proliferation (Ki67) and cell activity (CD69) and the production of cytokines such as IFN-γ and IL-2, impairing the anticancer function of normal CD8^+^ T cells, causing tumor progression (40). The research team further used microarray and functional enrichment analyses to identify 257 lncRNAs that actively participate in various processes regulating the activity of CD8^+^ T cells, such as metabolism, gene expression, and biosynthesis processes (40). Notably, in the above content, we demonstrated that CD8^+^ T cell-derived exosomes can activate CD8^+^ T cells with low affinity, which is contrary to the conclusion of this study. This can be attributed to the differences in CD8^+^ T cell subsets and activation states. In addition, CD8^+^ T cell-derived exosomes have been reported to be involved in directly promoting tumor progression, which is inconsistent with the function of the corresponding source cells. Exosomes from activated CD8^+^ T cells were shown to activate the ERK and NF-κB pathways in melanoma cells, leading to melanoma metastasis *in vivo* by increasing the expression of MMP9 *via* Fas signaling, suggesting a role for CD8^+^ T cell-derived exosomes in tumor immune escape (41). Owing to the dual role of CD8^+^ T cell-derived exosomes in tumor progression, tumor therapy strategies targeting exosomes need to consider the balancing mechanism involved.

### 3.2 CD4^+^ T cell-derived exosomes

T cells can be divided into various subsets based on their immunophenotypes, mainly CD4^+^ T helper cells and cytotoxic CD8^+^ T cells. CD4^+^ T cells can be further divided into Th1, Th2, Th9, Th17, Th22, follicular helper T cells, and regulatory T cells (Tregs), each of which produce specific effector cytokines under unique transcriptional regulation (42). CD4^+^ T cells interact with other cells, such as NK cells, macrophages, and CD8^+^ T cells, through the cytokines they produce. Shin et al. revealed that CD4^+^ T cell-derived exosomes increased the antitumor response of CD8^+^ T cells without affecting Tregs, thereby suppressing melanoma growth. Mechanistically, miR-25-3p, miR-155-5p, miR-215-5p, and miR-375 within CD4^+^ T cell-derived exosomes are responsible for inducing CD8^+^ T cell-mediated antitumor responses (43). This further supports the notion that exosomes are a novel form of CD8^+^ T cell activation by CD4^+^ T cells in addition to cytokines. However, the opposite was observed in another study, which suggested that exosomes released by CD4^+^ T cells inhibited CD8^+^ CTL responses and antitumor immunity in melanoma (44). It is worth considering whether this opposite result is caused by the heterogeneity of exosomes and whether there is a balancing mechanism.

In addition to influencing cellular immunity, CD4^+^ T cell-derived exosomes are involved in the regulation of humoral immunity (45). In this study, mice vaccinated with the hepatitis B surface antigen (HBsAg) vaccine showed a stronger humoral immune response to CD4^+^ T-cell-derived exosomes, indicating higher serum levels of hepatitis B surface antibody (HBsAb) (45). Additionally, CD4^+^ T cell-derived exosomes play an important role in B cell responses *in vitro*, which significantly promotes B cell activation, proliferation, and antibody production (45). It is well known that hepatitis B virus is the main cause of hepatocellular carcinoma (46–48), and the synergistic effect of CD4^+^ T cell-derived exosomes on HBsAb may contribute to the inhibition of hepatocellular carcinoma. Further research is required to confirm this hypothesis.

### 3.3 Treg-derived exosomes

Tregs are a group of lymphocytes that negatively regulate the immune response of the body and participate in tumor cells to evade immune surveillance (49). Owing to the significant immunosuppressive effects of Treg-derived exosomes, an increasing number of studies have focused on their role in tumor immune escape (50). Interestingly, Tregs have been reported to secrete more exosomes that express CD25, CTLA-4, and CD73 on the surface than other T cells. Exosomes expressing CD73 perform immunosuppressive functions by producing adenosine, which plays an important role in the anti-inflammatory response (51–53). In a recent phase I clinical trial, 18 patients with head and neck squamous cell carcinoma who received a combination of cetuximab, ipilimumab, and radiation therapy were serially monitored for Treg-derived exosomes, and Treg-derived exosomes were found to increase from the baseline levels (54), supporting the potential role of Treg-derived exosomes as non-invasive tumor and immune cell biomarkers in cancer. To promote clinical translation, researchers have further carried out relevant basic research. Tung et al. demonstrated for the first time that miRNAs, particularly miR-150-5p and miR-142-3p, are transferred from Tregs to DCs *via* Treg-derived exosomes, resulting in the production of a tolerogenic phenotype in DCs (55). Similarly, Xie et al. found that exosomes derived from natural CD8^+^ CD25^+^ Tregs significantly inhibited DC-induced CTL responses and antitumor immunity in a mouse B16 melanoma model (56). In addition to DCs, Treg-derived exosomes inhibit the expression of M1 macrophage markers and promote M2 macrophage markers (57). Macrophages are divided into classically activated M1 macrophages, which mainly exert anti-inflammatory and antitumor functions (58), and alternately activated M2 macrophages, which have immunosuppressive and tumor-promoting abilities (59). Therefore, induction of M2 macrophages by Treg-derived exosomes may promote tumor growth. Immunosuppression of Tregs mainly inhibits the activation and proliferation of CD4^+^ and CD8^+^ T cells (60). Studies have shown that exosomes derived from Tregs suppress T-cell proliferation (61, 62). In addition, Torri et al. revealed the inhibition of CD4^+^ T cell proliferation by Treg-derived exosomes (63). However, these studies have not yet confirmed the role of Treg-derived exosome-mediated immunosuppression of infiltrating T lymphocytes in tumor progression, which remains to be explored further.

## 4 T cell-derived exosomes in tumor immunotherapy

### 4.1 Engineered T cell-derived exosomes

Engineered exosomes mainly refer to modified exosomes with enhanced drug-loading efficiency, targeting, and resistance to body clearance after natural exosomes are treated with bioengineering techniques. Usually, the size and shape of these exosomes do not change significantly (64–67), but their membrane loaders or contents may differ significantly depending on the research purpose. Studies have shown that the clinical therapeutic effect of exosomes can be improved by changing their contents and surface substances to improve their targeting and drug-loading rate. For example, Lou et al. constructed an miR-199a-modified engineered exosome through genetic engineering and found that it could effectively transfer miR-199a to liver cancer cells. The miR-199a-modified engineered exosomes significantly increased the sensitivity of liver cancer cells to Adriamycin *in vitro*. It can also significantly promote the antitumor effect of Adriamycin in liver cancer *in vivo* (68). Another example is the loading of siRNA and oxaliplatin into bone marrow mesenchymal stem cell-derived exosomes *via* electroporation, which blocks the connection of tumor cells to macrophages, thus inhibiting the polarization of macrophages in the tumor microenvironment (69). Jung et al. generated IL-2-tethered exosomes from engineered Jurkat T cells expressing IL-2 at the plasma membrane *via* a flexible linker to induce an autocrine effect. Levels of miRNA in T cell-derived exosomes using IL-2 surface engineering were significantly altered, and differentially expressed miRNAs activated CD8^+^ T cells, enhancing their antitumor immune effects (27). Therefore, strengthening immune activity through engineering modification of CD4^+^ T cells and CD8^+^ T cell-derived exosomes is a novel strategy to improve the efficacy of tumor immunotherapy.

### 4.2 Depleting exosomes or blocking the uptake of exosomes

Given the role of Treg-derived exosomes and some CD8^+^ T cell-derived exosomes in tumor immune escape, depleting exosomes or blocking their uptake may be a novel cancer immunotherapy (70). The Aethlon ADAPT™ system, a novel device that can remove blood components below 200 nm, including exosomes that interact with the immobilized affinity agent of the device, was successfully applied for the first time in patients with hepatitis C virus. It could be speculated that if the Aethlon ADAPT™ system is used to eliminate immunosuppressive exosomes from T cells, it may improve the efficacy of antitumor immunotherapy.

### 4.3 Chimeric antigen receptor T cell-derived exosomes

Chimeric antigen receptor (CAR) T cells conjugate the antigen-binding part of an antibody that recognizes a tumor antigen and the intracellular part of the CD3-ζ chain or FcϵRIγ into a chimeric protein *in vitro*, and patient T cells are then transfected with gene transduction to express CAR (71). Patient T cells are “reprogrammed” to generate a large number of tumor-specific CAR-T cells, which have been successfully designed and used to treat malignant blood diseases (72). However, in the process of treating malignant tumors, CAR-T therapy inevitably has side effects such as cytokine release syndrome, neurotoxicity, and organ failure (73, 74). The management of CAR-T cell toxicity remains a challenge.

**Figure 2:**
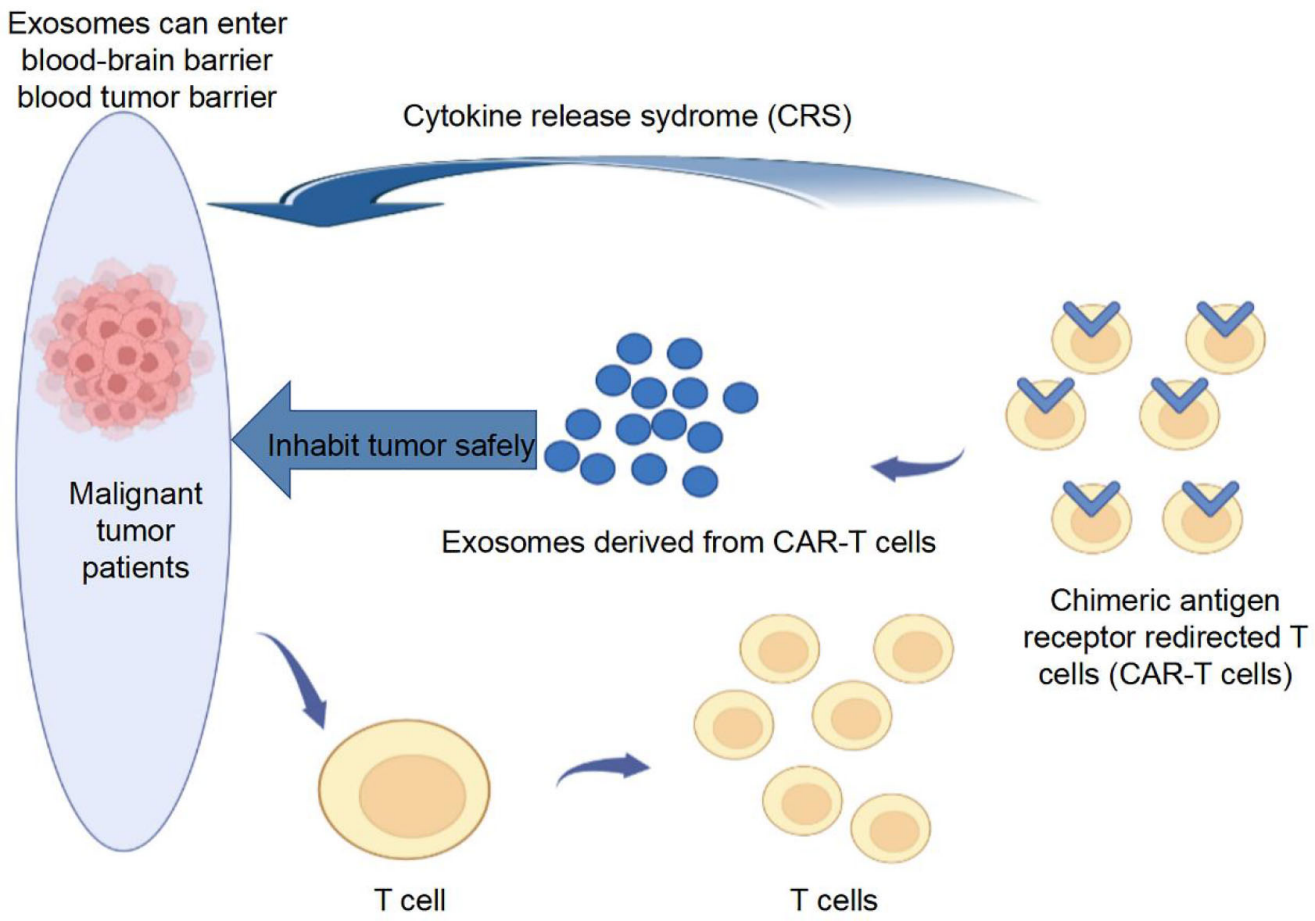
CAR-T cell-derived exosomes for tumor treatment. CAR-T cell-derived exosomes express high levels of cytotoxic molecule, making them effective vectors to provide pro-apoptotic cues to target tumor cells carrying homologous antigens.

CAR-T cell-derived exosomes have been reported to reduce the cytotoxicity of CAR-T therapy and cross the blood-brain barrier and blood-tumor barrier (13). CAR-T cell-derived exosomes express high levels of cytotoxic molecules (FasL, Apo2L, perforin, and grazyme A and B), making them effective vectors to provide pro-apoptotic cues to target tumor cells carrying homologous antigens (75). Several preclinical studies have confirmed that CAR-T cell-derived exosomes exert inhibitory effects on solid tumors, including TNBC and lung cancer, and are relatively safe (13, 76, 77). The mechanism of tumor apoptosis induced by CAR-T cell-derived exosomes is independent of FasL, Apo2L, perforin, and grazyme. A recent study demonstrated that CAR T cells contain RNA components of the tumor-suppressive signal-recognition particle 7SL1 (RN7SL1), a non-coding RNA that activates interferon-IFN stimulator genes (78). Notably, RN7SL1 is selectively transferred to immune cells *via* CAR-T cell-derived exosomes, restricting the development of bone marrow-derived suppressor cells and enhancing the immunostimulatory properties of DCs, thus effectively activating melanoma with endogenous CD8^+^ T cells that reject CAR antigens (78). Additionally, anticancer drugs can be loaded into exosomes from CAR-T cells to kill target tumor cells because of their excellent potential to penetrate the extracellular matrix of solid tumors (79). The above studies have shown that activated CAR-T cells can secrete exosomes to function in solid tumors and can affect the immune microenvironment of tumors; however, the current study seems to have failed to conclude whether CAR-T cell-derived exosomes play a role in hot or cold tumors (**Figure 2**).

## 5 Conclusions

This review summarized the role of CD8^+^ T cells, CD4^+^ T cells, and Treg-derived exosomes in tumor immune modulation and revealed the potential application of T cell-derived exosomes in tumor immunotherapy, including engineered T cell-derived exosomes, depleting exosomes, or blocking the uptake of exosomes and CAR-T cell-derived exosomes. However, studies on T cell-derived exosomes remain in the exploratory stage. There are still many hurdles to overcome before T cell-derived exosomes can transition from the laboratory to the clinic. First, the purification and characterization methods of exosomes vary from laboratory to laboratory, and different methods may confuse the subgroups and physicochemical properties of exosomes. Therefore, researchers need to refer to the International Society of Extracellular Vesicles and the standardization efforts for exosome isolation, purification, and use for therapeutics. The second problem is exosome production. The number of exosomes extracted from cells is small and it is often difficult to meet the requirements of drug delivery. Therefore, to continue expanding the applications of exosomes, a large-scale production mode is needed. In addition, the stability and toxicity of exosomes after modification or drug loading need to be further explored, especially as vectors for tumor nanomedical applications. These findings will facilitate clinical transformation of exosomes (80). Additionally, the best exosome therapy candidate payload is currently inconclusive and needs to be explored further in the future.

Exosomes have many advantages over other drug delivery systems, especially their high stability, low immunogenicity, ability to avoid clearance by mononuclear phagocytes, good biocompatibility, high bioactivity, and high targeting efficiency. We believe that with the joint efforts of immunologists, molecular biologists, chemists, and physicians, T cell-based exosomes will become a powerful tool in the fight against tumors in the future.

## Author contributions

QZ and SW wrote the manuscript and created the figures. HW, YL and SF collected and prepared the related papers. YC and CW conceived the final approval of the version to be submitted and obtaining of the funding. All authors read and approved the final manuscript.
